# Acute hyperammonemic encephalopathy after fluoropyrimidine-based chemotherapy: A case series and review of the literature

**DOI:** 10.1097/MD.0000000000006874

**Published:** 2017-06-02

**Authors:** Seiichiro Mitani, Shigenori Kadowaki, Azusa Komori, Keiji Sugiyama, Yukiya Narita, Hiroya Taniguchi, Takashi Ura, Masashi Ando, Yozo Sato, Hidekazu Yamaura, Yoshitaka Inaba, Makoto Ishihara, Tsutomu Tanaka, Masahiro Tajika, Kei Muro

**Affiliations:** a Department of Clinical Oncology; b Department of Diagnostic and Interventional Radiology; c Department of Endoscopy, Aichi Cancer Center Hospital, Nagoya, Japan.

**Keywords:** 5-fluorouracil, capecitabine, fluoropyrimidines, hyperammonemic encephalopathy, S-1, sarcopenia

## Abstract

Acute hyperammonemic encephalopathy induced by fluoropyrimidines (FPs) is a rare complication. Its pathophysiology remains unclear, especially given the currently used regimens, including intermediate-doses of 5-fluorouracil (5-FU) or oral FP agents. We aimed to characterize the clinical manifestations in cancer patients who developed hyperammonemic encephalopathy after receiving FP-based chemotherapy.

We retrospectively reviewed 1786 patients with gastrointestinal or primary-unknown cancer who received FP-based regimens between 2007 and 2012. Eleven patients (0.6%) developed acute hyperammonemic encephalopathy. The incidence according to the administered anticancer drugs were as follows: 5-FU (8 of 1176, 0.7%), S-1 (1 of 679, 0.1%), capecitabine (2 of 225, 0.9%), and tegafur-uracil (UFT) (0 of 39, 0%). Ten patients (90.9%) had at least 1 aggravating factor, including infection, dehydration, constipation, renal dysfunction, and muscle loss. All the 10 patients met the definition of sarcopenia. Median time to the onset of hyperammonemic encephalopathy in the cycle was 3 days (range: 2–21). Three patients (27.3%) developed encephalopathy during the first cycle of the regimen and the remaining 8 patients during the second or more cycles. Seven patients (63.6%) had received at least 1 other FP-containing regimen before without episodes of encephalopathy.

All patients recovered soon after immediate discontinuation of chemotherapy and supportive therapies, such as hydration, infusion of branched-chain amino acids, and oral lactulose intake, with a median time to recovery of 2 days (range: <1–7). Four patients (36.4%) received FP-based regimens after improvement of symptoms; 3 patients were successfully managed with dose reduction, and 1 patient, who had developed encephalopathy due to S-1 monotherapy, received modified FOLFOX-6 therapy without encephalopathy later.

FP-associated acute hyperammonemic encephalopathy is extremely rare, but a possible event at any time and even during the administration of oral FP agents. Particular attention is warranted when giving FP-based therapy for patients with aggravating factors, such as sarcopenia. This complication can be properly managed with early detection.

## 1 Introduction

Fluoropyrimidines (FPs), which include 5-fluorouracil (5-FU) and its oral prodrugs S-1, capecitabine, and tegafur-uracil (UFT), have been prescribed for various malignancies involved in the gastrointestinal tract, breast, head and neck, and ovaries.^[1–3]^ Common adverse events of FPs include bone marrow toxicities, anorexia, diarrhea, mucositis, and hand-foot syndrome. Acute hyperammonemic encephalopathy secondary to FPs is a rare complication that generally occurs after infusion of high-dose 5-FU, and its pathophysiology remains unclear. The incidence of encephalopathy induced by 5-FU has been reported to be 5.7% to 8.7% among patients treated with high dose of 5-FU infusion (2600 mg/m^2^ over 24 hours, every week).^[4,5]^ As for currently used regimens, including intermediate dose of 5-FU infusion or oral FP, this toxicity has been described only in case reports or case series.^[6–16]^ Hence, incidence and optimal management are not fully understood. Most cases completely and rapidly recover, but clinical features in some cases can be fatal.^[15–17]^ Therefore, it is important for clinicians to recognize this complication early and treat appropriately. The aim of this study was to estimate the incidence of hyperammonemic encephalopathy and to characterize the clinical manifestations in cancer patients who developed hyperammonemic encephalopathy after receiving widely used FP-based chemotherapy.

## 2 Methods

### 2.1 Patients

We retrospectively reviewed a computerized database of 1786 patients with gastrointestinal or primary-unknown cancer who had locally advanced and/or metastatic lesions and received FP-based regimens at the Aichi Cancer Center Hospital between January 2007 and December 2012. FP antitumor drugs included 5-FU, S-1, capecitabine, and UFT. Among these patients, we identified patients who developed acute hyperammonemic encephalopathy after receiving FP-based chemotherapies. This study was approved by the Institutional Review Board at Aichi Cancer Center Hospital.

### 2.2 Definitions

Acute hyperammonemic encephalopathy was diagnosed according to previously reported criteria: development of encephalopathy during or shortly after completion of FPs administration; exclusion of other metabolic or physical factors that may have an effect on the consciousness level; and exclusion of a drug effect by concomitant medications.^[4]^ Additionally, patients with hepatic failure due to liver metastases or coexisting liver cirrhosis were excluded.

### 2.3 Data collection

Patient demographics obtained from medical records were as follows: the Eastern Cooperative Oncology Group performance status (ECOG PS), primary tumor location, histology, chemotherapeutic regimen, time from the start of chemotherapy to onset of encephalopathy, and survival time. Data on infection, dehydration, constipation, renal dysfunction, and muscle loss, which were proposed as risk factors for hyperammonemic encephalopathy by Liaw et al^[6]^ and Kikuta et al^[10]^ were also collected. Status of patients was evaluated at the time of the initiation of course when patients developed encephalopathy. Muscle loss was evaluated using the skeletal muscle index (SMI), which was calculated via a previously described method using a cross-sectional computed tomography image of the third lumbar vertebra (L3).^[18]^ Patients were considered to have sarcopenia, a status of skeletal muscle loss, when SMI was lower than 34.9 cm^2^/m^2^ for women and 40.8 cm^2^/m^2^ for men.^[19]^ Encephalopathy was assessed by depressed level of consciousness (Common Terminology Criteria for Adverse Events version 4.0). Survival was defined as the time from the diagnosis of hyperammonemic encephalopathy to death. Probabilities for survival were determined using the Kaplan–Meier method.

## 3 Results

### 3.1 Patients

Of the 1786 patients, 1176 (65.8%), 679 (38.0%), 255 (14.3%), and 39 (2.2%) patients received 5-FU, S-1, capecitabine, and UFT, respectively. None of the patients received infusion 5-FU on a high-dose schedule (>2000 mg/m^2^ over 24 hours). Primary tumor sites were as follows: colorectal (n = 723), gastric (n = 618), esophageal (n = 361), unknown (n = 55), anal (n = 16), and small bowel (n = 13). Hyperammonemic encephalopathy occurred in 13 patients (0.7%) during chemotherapy. Of them, 2 patients were excluded for the following reasons: each patient had hepatic failure due to multiple liver metastases and multiple liver metastases with coexisting alcoholic cirrhosis, respectively. Accordingly, 11 patients (0.6%) were identified as developing acute hyperammonemic encephalopathy. The incidence according to the administered anticancer drugs were as follows: 5-FU (8 of 1176, 0.7%), S-1 (1 of 679, 0.1%), capecitabine (2 of 225, 0.9%), and UFT (0 of 39, 0%).

The baseline patient characteristics are shown in Table 1. The median age was 62 years (range: 38–75). All patients had metastatic lesions. Five patients (45.5%) had poor PS (ECOG PS ≥2). The rate of occurrence by primary site was as follows: gastric (5 of 618, 0.8%), colorectal (4 of 723, 0.6%), esophagus (1 of 361, 0.3%), and unknown primary (1 of 55, 1.8%). Seven patients (63.6%) had received at least 1 other regimen before. Among these patients, all 7 patients received another regimen containing FPs, and 3 patients had previously received a regimen containing the same FP agent. Ten patients (90.9%) had at least 1 predisposing factor: all except 1 were diagnosed with sarcopenia.

**Table 1:**
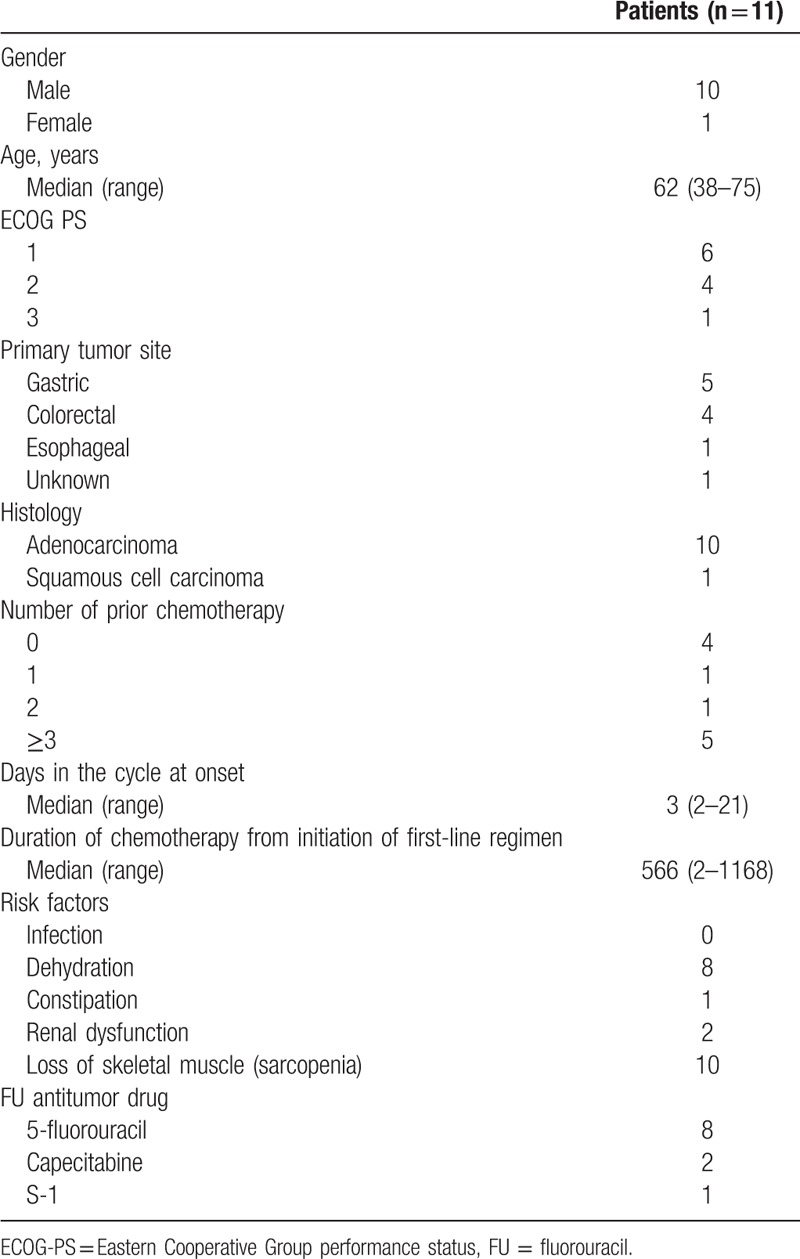
Demographic and clinical characteristics in patients developing hyperammonemic encephalopathy.

### 3.2 Clinical course

The clinical courses for the 11 patients are shown in Table 2. Three patients developed encephalopathy during the first cycle of the regimen and the remaining 8 patients during the second or more cycles. Median time to the onset of hyperammonemic encephalopathy in the cycle was 3 days (range: 2–21). While the onset of symptoms was observed during or shortly after exposure to FPs for 9 patients, 2 patients who received oral FP agents developed encephalopathy on days 15 and 21 of the cycle, respectively. Median time from initiation of first-line chemotherapy to the onset was 567 days (range: 3–1169). Case 10 developed encephalopathy in the 26th cycle of simplified LV5FU2 after 978 days from the initiation of regimen. All the patients presented with altered mental status: 2 patients (18.1%) developed Grade 1 depressed consciousness; 6 patients (54.5%) developed Grade 2; and 3 patients (27.2%) developed Grade 3. The median level of serum ammonia was 253 μg/dL (range: 213–> 400).

**Table 2:**
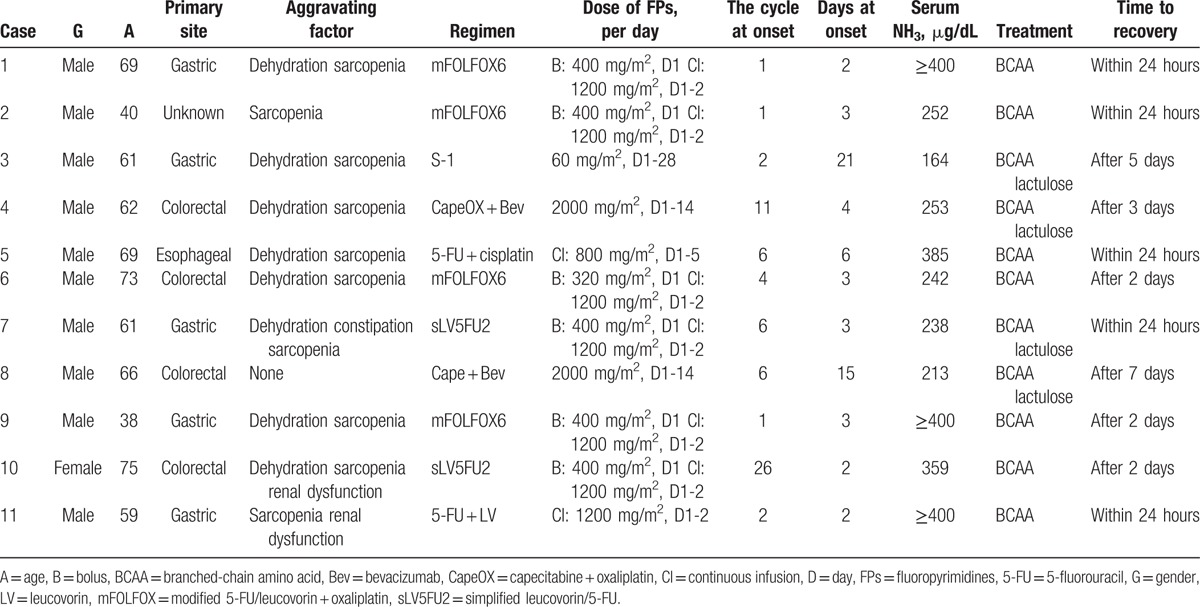
Treatment and clinical courses.

**Figure 1:**
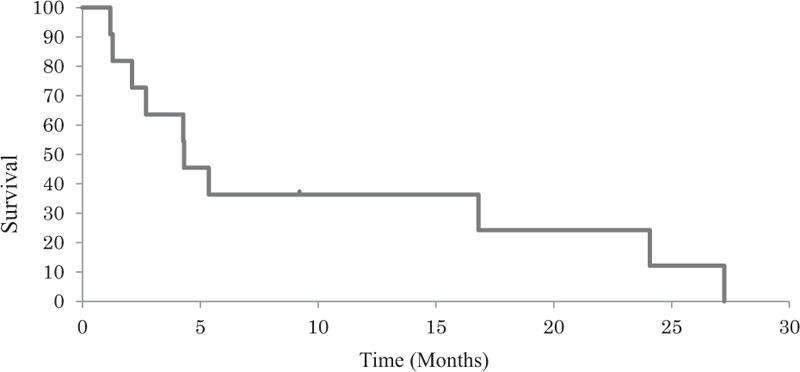
Kaplan–Meier curves are shown for overall survival after hyperammonemic encephalopathy.

If administration of FPs was ongoing, chemotherapy was stopped immediately. Adequate fluid infusion, including branched-chain amino acids, was administrated for all patients, and 4 patients took lactulose orally. Immediate improvement of symptoms was obtained, followed by normalization of ammonia levels within 1 week. After the improvement of symptoms, 6 patients (Cases 2, 3, 5, 8, 9, and 10) resumed chemotherapy and best supportive care was selected for the remaining 5 patients. Among the 6 patients who received chemotherapy, 3 patients (Cases 8, 9, and 10) continued the same regimen after recovery from encephalopathy. Cases 9 and 10 were successfully managed with 50% dose reduction of 5-FU continuous infusion until disease progression. Case 8 continued the same dose of capecitabine as symptoms were relatively mild. Although mild hyperammonemic encephalopathy recurred with slightly elevated level of serum ammonia and dose reduction of capecitabine was performed eventually, a total of 8 cycles were delivered until disease progression. Case 3, who had developed encephalopathy due to S-1 monotherapy, received modified FOLFOX-6 without encephalopathy later. The median overall survival time after hyperammonemic encephalopathy was 4.3 months (Fig. 1). Although most patients died soon after the development of acute hyperammonemic encephalopathy, 3 patients, all of whom had resumed chemotherapy, survived for more than 1 year.

## 4 Discussion

In this study, we identified 1786 patients treated with widely used FP-based chemotherapies over the study period, with acute hyperammonemic encephalopathy occurring in 11 (0.6%). The overall incidence was much lower than that reported for patients on high-dose 5-FU (5.7% and 8.7%, respectively).^[4,5]^ This is probably explained by the dose of 5-FU. In the study by Liaw et al,^[6]^ high daily dose of 5-FU (1800  or 2600 mg/m^2^) was significantly associated with higher plasma ammonia levels and more rapid onset of hyperammonemic encephalopathy (mean: 3.2 vs 1.1 days, *P*<.0001) compared with a low daily dose (500 or 1000 mg/m^2^). Our finding also highlights the importance of recognizing this toxicity even in the use of oral FP agents. Intriguingly, the estimated incidence in S-1 (0.1%) appeared lower than that in 5-FU infusion (0.7%). This difference may be attributable to the effect of dihydropyrimidine dehydrogenase inhibition that usually leads to the decrease of 5-FU catabolites.

Although the precise mechanism of hyperammonemic encephalopathy remains to be fully understood, it was proposed by Koenig and Patel^[20]^ that fluoroacetate, one of the catabolites of 5-FU, directly suppresses the Krebs cycle and in turn causes dysfunction in the ATP-dependent urea cycle, leading to transient accumulation of ammonium. In general, ammonium produced through 5-FU catabolism itself does not cause hyperammonemia, but infection, dehydration, constipation, and renal dysfunction aggravate this condition.^[6]^ In addition, body weight loss accompanied by muscle atrophy can promote accumulation of ammonium as the skeletal muscle is an important site of ammonia catabolism.^[10]^ Most of the patients in our cohort had at least 1 predisposing factor similar to factors outlined in previous reports,^[6,8–11,15]^ and all 7 patients in our study with a previous exposure to FPs experienced no episode of encephalopathy. It is noteworthy that most of patients (90.9%) met the definition of sarcopenia. Although body mass index was also examined, 7 patients (63.6%) were within normal limits (18.5–< 22.5). Previous reports concluded that sarcopenia is associated with toxicities of chemotherapy.^[21–24]^ Our results suggested that sarcopenia can also be a predictive marker for the development of hyperammonemic encephalopathy. The majority of patients distinctively had multiple lines of therapies and poor ECOG PS, resulting in worse prognosis.

Our findings were similar to those in previous reports on 5-FU-induced encephalopathy with respect to the time to onset and the time to recovery. All patients developed encephalopathy during or shortly after 5-FU exposure (range: 2–6 days) and recovered with the decrease of serum ammonia within 3 days after immediate discontinuation of FPs and supportive therapies, consistent with existing reports.^[6–9,12]^ In a large cohort study of 29 patients with 5-FU-related encephalopathy, the time to onset ranged from 0.5 to 5 days.^[6]^ Most of the patients recovered within 2 days, but 4 patients with infection remained comatose. In this study, bacterial infection was associated with higher plasma ammonium concentrations and more rapid onset of hyperammonemia^[6]^; therefore, no comorbid infectious disease in our series may account for the observed good prognosis. The number of cycles or lines at the onset of encephalopathy varied. Of the 11 patients, only 4 (36.3%) experienced the events at the first exposure to the FP drug. Among 3 patients receiving oral FP agents, 2 developed encephalopathy on days 21 and 15 of the cycle. Kwon et al^[14]^ reported a case of hyperammonemic encephalopathy developed 14 days after the completion of 5-FU infusion. Therefore, clinicians should be aware of this entity whenever FP-containing regimens are continued despite the number of cycles or lines of chemotherapy.

There is a lack of reliable data informing and providing guidance for alternative treatments for patients experiencing FP-associated encephalopathy. In some reports, reducing the dose of infusion 5-FU or exchanging it with capecitabine chemotherapy was successfully done without recurrence.^[7–9]^ In the study by Liaw et al,^[6]^ 7 of 29 patients received the same regimen after treating hyperammonemic encephalopathy without recurrence. In our series, 3 patients (27.3%) received another 2 or more cycles of the same regimen with dose reduction and were successfully re-challenged. One patient with a previous history of encephalopathy due to S-1 monotherapy continued modified FOLFOX-6 therapy successfully. Regarding continuing chemotherapy, not only the re-challenge of the same regimen with dose reduction but also switching to another FPs regimen can be a reasonable option.

There are several limitations in our study. First, it was difficult to confirm whether patients developed hyperammonemic encephalopathy as a result of FPs only. Five patients (45.4%) received a regimen in combination with oxaliplatin. However, there are some reports regarding FP-induced hyperammonemic encephalopathy, while there is only 1 case report that was suspected to develop hyperammonemic encephalopathy due to oxaliplatin use.^[25]^ Second, we did not check the level of ammonia in all patients who received FPs. Patients with mild symptoms could be overlooked, and the incidence may be underestimated. However, this is the first systematic report to show the clinical features of FP-induced hyperammonemic encephalopathy in currently used FP-based regimens despite these limitations.

In summary, acute hyperammonemic encephalopathy is rare but warrants much attention given the toxicity of FP-based chemotherapy, which remains to be the treatment cornerstone of various solid cancers. Clinicians should be aware of this entity in every treatment cycle and even in the administration of oral FP agents, especially in the presence of possible predisposing factors. Nonetheless, this complication can be effectively treated due to early detection and proper management.

## References

[1] McKendrick J Coutsouvelis J Capecitabine: effective oral fluoropyrimidine chemotherapy. Expert Opin Pharmacother 2005;6:1231–9. 15957975 10.1517/14656566.6.7.1231

[2] Longley DB Harkin DP Johnston PG 5-fluorouracil: mechanisms of action and clinical strategies. Nat Rev Cancer 2003;3:330–8. 12724731 10.1038/nrc1074

[3] Saif MW Syrigos KN Katirtzoglou NA S-1: a promising new oral fluoropyrimidine derivative. Expert Opin Investig Drugs 2009;18:335–48. 10.1517/13543780902729412 19243284

[4] Yeh KH Cheng AL High-dose 5-fluorouracil infusional therapy is associated with hyperammonaemia, lactic acidosis and encephalopathy. Br J Cancer 1997;75:464–5. 10.1038/bjc.1997.79 PMC2063385 9020500

[5] Lin YC Chen JS Wang CH Weekly high-dose 5-fluorouracil (5-FU), leucovorin (LV) and bimonthly cisplatin in patients with advanced gastric cancer. Jpn J Clin Oncol 2001;31:605–9. 11902492 10.1093/jjco/hye130

[6] Liaw CC Wang HM Wang CH Risk of transient hyperammonemic encephalopathy in cancer patients who received continuous infusion of 5-fluorouracil with the complication of dehydration and infection. Anticancer Drugs 1999;10:275–81. 10327032 10.1097/00001813-199903000-00004

[7] Yi HJ Hong KS Moon N Acute hyperammonemic encephalopathy after 5-fluorouracil based chemotherapy. Ann Surg Treat Res 2016;90:179–82. 26942162 10.4174/astr.2016.90.3.179 PMC4773463

[8] Thomas SA Tomeh N Theard S Fluorouracil-induced hyperammonemia in a patient with colorectal cancer. Anticancer Res 2015;35:6761–3. 26637893

[9] Advani PP Fakih MG 5-FU-induced hyperammonemic encephalopathy in a case of metastatic rectal adenocarcinoid successfully rechallenged with the fluoropyrimidine analog, capecitabine. Anticancer Res 2011;31:335–8. 21273620

[10] Kikuta S Asakage T Nakao K The aggravating factors of hyperammonemia related to 5-fluorouracil infusion—a report of two cases. Auris Nasus Larynx 2008;35:295–9. 17826933 10.1016/j.anl.2007.04.012

[11] Cheung WY Fralick RA Cheng S The confused cancer patient: a case of 5-fluorouracil-induced encephalopathy. Curr Oncol 2008;15:234–6. 19008998 10.3747/co.v15i5.252 PMC2582518

[12] Kim YA Chung HC Choi HJ Intermediate dose 5-fluorouracil-induced encephalopathy. Jpn J Clin Oncol 2006;36:55–9. 16436463 10.1093/jjco/hyi214

[13] Kim SR Park CH Park S Genetic polymorphisms associated with 5-fluorouracil-induced neurotoxicity. Chemotherapy 2010;56:313–7. 20714149 10.1159/000320032

[14] Kwon KA Kwon HC Kim MC A case of 5-fluorouracil induced encephalopathy. Cancer Res Treat 2010;42:118–20. 20622967 10.4143/crt.2010.42.2.118 PMC2901079

[15] Martinez-Lapiscina EH Erro ME Cabada T 5-Fluorouracil induced hyperammonemic encephalophathy: etiopathologic correlation. Can J Neurol Sci 2012;39:553–4. 22896877

[16] Lazier J Lupichuk SM Sosova I Hyperammonemic encephalopathy in an adenocarcinoma patient managed with carglumic acid. Curr Oncol 2014;21:e736–9. 25302046 10.3747/co.21.2076 PMC4189581

[17] Lukaschek J Nufer M Maurer D Cardiotoxicity and neurotoxicity of high-dose continuous fluorouracil as a result of degradation compounds in the drug vials. J Clin Oncol 2004;22:5022–5. 15611524 10.1200/JCO.2004.04.272

[18] Choi Y Oh DY Kim TY Skeletal muscle depletion predicts the prognosis of patients with advanced pancreatic cancer undergoing palliative chemotherapy, independent of body mass index. PLoS One 2015;10:e0139749. 26437072 10.1371/journal.pone.0139749 PMC4593598

[19] Zhuang CL Huang DD Pang WY Sarcopenia is an independent predictor of severe postoperative complications and long-term survival after radical gastrectomy for gastric cancer: analysis from a large-scale cohort. Medicine (Baltimore) 2016;95:e3164. 27043677 10.1097/MD.0000000000003164 PMC4998538

[20] Koenig H Patel A Biochemical basis for fluorouracil neurotoxicity. The role of Krebs cycle inhibition by fluoroacetate. Arch Neurol 1970;23:155–60. 5430334 10.1001/archneur.1970.00480260061008

[21] Strulov Shachar S Deal AM Weinberg M Skeletal muscle measures as predictors of toxicity, hospitalization, and survival in patients with metastatic breast cancer receiving taxane based chemotherapy. Clin Cancer Res 2016;23:658–65. 27489287 10.1158/1078-0432.CCR-16-0940 PMC5290138

[22] Prado CM Baracos VE McCargar LJ Sarcopenia as a determinant of chemotherapy toxicity and time to tumor progression in metastatic breast cancer patients receiving capecitabine treatment. Clin Cancer Res 2009;15:2920–6. 19351764 10.1158/1078-0432.CCR-08-2242

[23] Tamandl D Paireder M Asari R Markers of sarcopenia quantified by computed tomography predict adverse long-term outcome in patients with resected oesophageal or gastro-oesophageal junction cancer. Eur Radiol 2016;26:1359–67. 26334504 10.1007/s00330-015-3963-1

[24] Blauwhoff-Buskermolen S Versteeg KS de van der Schueren MA Loss of muscle mass during chemotherapy is predictive for poor survival of patients with metastatic colorectal cancer. J Clin Oncol 2016;34:1339–44. 26903572 10.1200/JCO.2015.63.6043

[25] Chang YY Lin JK Jiang JK Oxaliplatin-related hyperammonaemic encephalopathy in a patient with colon cancer. Colorectal Dis 2012;14:e821. 22330089 10.1111/j.1463-1318.2012.02986.x

